# Comparison of Autistic Individuals Who Engage in Self-Injurious Behavior, Aggression, and Both Behaviors

**DOI:** 10.3390/pediatric13040066

**Published:** 2021-10-01

**Authors:** Stephen M. Edelson

**Affiliations:** Autism Research Institute, San Diego, CA 92116, USA; director@autism.org

**Keywords:** aggression, self-injurious behavior, self-harming behaviors, challenging behaviors, medical comorbidities, autism spectrum disorder

## Abstract

Background. Two of the most challenging behaviors exhibited by individuals on the autism spectrum are self-injurious behavior (SIB) and aggression. The aim of this study was to identify co-occurring symptoms, behaviors, and medical comorbidities that may provide insight into understanding and treating these behaviors. Method. A large-scale online survey was used to collect data on symptoms, behaviors, and medically related comorbidities commonly reported in individuals with autism spectrum disorders (ASD). Based on responses from 2327 participants, individuals with ASD were divided into four categories: individuals who engaged in SIB only, individuals who engaged in aggression only, individuals who engaged in both behaviors, and individuals who engaged in neither behavior. Results. There were several characteristics and comorbidities associated with those who engaged in SIB only and in aggression only, in addition to those who engaged in both behaviors. Conclusion. The findings in this study provide evidence to support at least two underlying causes of these behaviors (insensitivity to pain and reactions to food) as well as implications for treating them. Furthermore, several behaviors often observed during early childhood may be considered early predictors of these challenging behaviors.

## 1. Introduction

Severe behaviors directed towards oneself or others have been discussed for over 50 years in the autism literature [1,2]. Researchers have studied the profiles of individuals who engage in these behaviors in order to better understand the underlying reasons, and to develop preventive strategies and treatments [3].

Self-injurious behavior (SIB) is one of the most devastating behaviors exhibited by individuals with intellectual disabilities. SIB often leads to some form of tissue damage, such as redness, bruises, lacerations, and, in severe cases, bone fractures. Examples include ear hitting, hand biting, hair pulling, head banging, and excessive rubbing and scratching. Aggressive behaviors can lead to injuries to others and typically involve hitting, biting, and hair pulling. In general, research has shown that those who engage in SIB have more severe adaptive deficits and intellectual disabilities than those who exhibit aggression [4,5].

Both challenging SIB and aggression have been associated with medical comorbidities including anxiety, gastrointestinal (GI) distress, sensory sensitivities, and sleep disturbances [6,7]. In addition, the behavioral literature has demonstrated that social interactions may trigger and/or reinforce these behaviors [8,9].

In this study, parents, relatives, therapists, and several individuals with ASD completed an extensive survey designed to classify individuals with ASD based on symptoms, behavioral characteristics, and medical comorbidities. Results were analyzed in relation to those who engaged in only SIB, only aggression, both behaviors, and neither behavior.

## 2. Method

### 2.1. Evaluation Structure

Two questionnaires were employed in this study. The Diagnostic Checklist Form E-2 was developed by researcher Bernard Rimland and has been widely used in autism research over the past five decades. A second, more recent questionnaire, consisting mostly of questions on medical comorbidities, was developed with the participation of several leading researchers. This questionnaire addressed allergies, anxiety, gastrointestinal disease, sensory sensitivities, and sleep disturbances.

### 2.2. Demographics

The introductory instructions to the survey stated that all participants had to have received a diagnosis of autism spectrum disorder. A total of 82% of the cases were males, and 16.5% were females. The male-to-female ratio is consistent with the 4:1 ratio documented by the Autism and Developmental Disabilities Monitoring Network funded by the Centers for Disease Control and Prevention [10].

Age was classified into five categories. A total of 3% of cases were under three years of age; 7.3% were between the ages of three and four years of age; 7.4% were between the ages of four and five years of age; 10.1% were between the ages of five and six years of age; and 72.1% were over six years of age.

### 2.3. Collection of Evaluations

The two questionnaires were uploaded to an Internet survey service (Alchemer.com, accessed on 9 September 2017), and the online program was HIPAA-compliant. Participants were recruited from the Autism Research Institute’s website (autism.com, accessed on 9 September 2017) and several e-newsletters published from 2015 to 2017.

A total of 2327 individuals completed the survey. This included 1966 mothers, 251 fathers, 82 relatives (e.g., aunts, grandparents, siblings, step-parents), 9 therapists, and 19 individuals on the autism spectrum.

## 3. Data Analysis

Four categories were investigated. These included SIB only, aggression only, SIB plus aggression, and neither SIB nor aggression. The response tallies were calculated by an asp.net program that queried a Microsoft Access database. Since the responses were categorical, a non-parametric statistical test, i.e., chi-square, was utilized. An online program calculated the chi-square values and probability levels (Social Science Statistics, www.socscistatistics.com, accessed on 10 July 2021). The alpha level was set at *p* < 0.5. A Bonferroni correction for multiple comparisons was calculated into the analyses. The alpha level was *p* = 0.0029.

Not all users answered every question on the two checklists. With regard to the questions under investigation, the percentage of missing data ranged from 1.8% to 2.6%.

## 4. Procedure

A limited number of responses to certain questions were selected prior to the analyses and were based primarily on previous research. These included responses pertaining to commonly reported behavioral characteristics (e.g., destructiveness, rocking), medical comorbidities, (e.g., gastrointestinal health, seizures), and sensory sensitivities (e.g., auditory, visual). Early signs of impairment were also studied in order to identify possible predictors of these behaviors (e.g., receptive language, rocking).

## 5. Results

Analyses of sex and age differences are presented in Table 1. There were no significant differences with respect to sex and age with regard to SIB only, aggression only, exhibiting both behaviors, and neither behavior (*χ*^2^ = 9.09, *p* > 0.05 and *χ*^2^ = 25.34, *p* > 0.05, respectively).

There were no specific symptoms, behaviors, or medical comorbidities unique to those individuals who engaged in SIB only or aggression only. However, certain symptoms and behaviors occurred to a significant degree both in individuals who engaged in SIB without aggression and in individuals who engaged in both SIB and aggression (see Table 2). These included “suffered from at least one seizure as a child”, “suspected low intelligence”, “does not feel normal levels of pain”, “sleeps less than eight hours in a 24-h period”, “often anxious or nervous”, “sometimes whirls himself like a top”, and “little or no receptive language”.

In addition, individuals who exhibited both SIB and aggression engaged in destructive behavior (See Table 3). They were also more likely to become upset when certain things were not “right”.

There were two additional responses that were more likely to occur in those who engaged in either one or both behaviors (See Table 4). These individuals were more likely to rock in their crib as a baby and to react to certain foods.

Interestingly, those suffering from GI distress tended not to engage in SIB or aggression. This was true for both constipation and diarrhea. See Table 5.

Other specific responses to questions were relatively common among all cases and were not statistically significant. These included auditory and/or visual hypersensitivities (*χ*^2^ = 8.6206, *p* > 0.05) and poor coordination (*χ*^2^ = 0.1810, *p* > 0.05).

## 6. Discussion

There were no differences between the sex of the individuals and age with respect to the four categories under investigation (i.e., SIB only, aggression only, both behaviors, neither behavior). Several symptoms and behaviors as well as medical comorbidities were more prevalent in those who engaged in SIB and aggression than those who did not engage in either behavior. There were no characteristics that were much more likely to occur with those who engaged in only SIB or only aggression.

Table 6 contains a summary of the results.

Numerous symptoms, behaviors, and medical comorbidities were associated with both individuals who engaged in SIB only and those who engaged in SIB and aggression. (Note: All of these individuals engaged in some form of SIB, but not all of them exhibited aggression.) Many of these characteristics and comorbidities have been reported in the SIB literature. They include rocking (repetitive/ritualistic behaviors) [5]; seizures [11], impaired cognitive ability [12], anxiety or nervousness [13], and sleep problems [14].

Furthermore, 67% of individuals were reported to lack pain sensations. It is possible that a significant number of these individuals may benefit from naltrexone, an opioid antagonist, which has received a reasonable amount of experimental support [15]. Sandman and colleagues theorized that physical trauma to the body from SIB may release endogenous endorphins [16,17]. Endorphins, in general, lead to a feeling of pleasure or euphoria in addition to dulling or eliminating pain from the behavior. Since naltrexone blocks the release of endorphins, its use causes SIB to be perceived as painful and no longer rewarding.

A number of individuals with ASD exhibited both SIB and aggression. This may be interpreted as a generalized aggressive response with no preference or inclination for either form of behavior. A thorough analysis of the behavior, such as a functional behavior assessment or FBA, would likely be able to determine the reason for the two behaviors [9]. Cases in which both behaviors serve the same purpose or function would be consistent with this interpretation.

Individuals who exhibited both SIB and aggression also exhibited destructive behaviors, and they tended to be upset when certain things changed. An FBA would likely be able to determine whether these behaviors typically occur together or separately, and whether they serve similar or different functions.

It is also important to mention that those individuals who exhibited either one or both behaviors were reported to be more reactive to foods than those who did not engage in either behavior. Food allergies and food sensitivities in individuals with ASD have often been reported by experienced clinicians [18,19]. Studies have also documented the impact of food allergies and sensitivities on several biological systems in relation to challenging behaviors [7,20]. A better understanding of how food allergies and sensitivities are directly or indirectly associated with challenging behaviors is very much needed in order to design effective treatments and methods of preventing these behaviors.

Unexpectedly, those who did not engage in either SIB or aggression were more likely to experience GI problems such as constipation and diarrhea. Other researchers have reported a relationship between GI issues and challenging behaviors [7,21], whereas Maenner, Arneson, Levy, Kirby, and Nicholas et al. [22] reported that aggressive behaviors were common in those both with and without GI problems in a large-scale study of children with ASD.

Researchers have reported that challenging behaviors in ASD may start at an early age but can also begin later in life [4,23]. Some of the characteristics noted in this investigation may be early predictors of challenging behaviors, and caregivers could be told to look for such signs in order to reduce or eliminate behavior problems before they may become severe and difficult to manage later in life. These early characteristics included “engages in stereotypic, repetitive behaviors (rocking behavior)”, “exhibits destructive behaviors”, “has experienced a seizure”, “has little or no receptive language”, “indications of low intelligence”, “is upset when certain things are ‘not right,’” and “whirls like a top”.

## 7. Limitations

The limitations in this study are inherent in many, if not most, online surveys. All respondents had to have access to the Internet. In addition, survey respondents’ recall of past events is not always perfect. However, many of the issues under investigation are considered troubling and may be more likely to be remembered [24]. There were also some missing data, ranging from 1.8% to 2.6% per question. Users may have purposely or inadvertently skipped these questions.

Measurement of other variables may have helped explain some of the variability within the data. This includes age differences of those individuals over six years, the frequency and severity of their symptoms and behaviors, and the extent of their medical co-morbidities and sensory sensitivities.

## 8. Conclusions

Overall, the results of this study are consistent with previous research. Numerous symptoms, behaviors, and medical comorbidities were more likely to occur in individuals with ASD who engaged in either one or both challenging behaviors as compared to those who did not exhibit either one. Inquiries about certain characteristics and comorbidities, such as sensitivity to pain and reactions to foods, may help guide treatment efforts. Finally, possible early signs may point to appropriate interventions that can help to circumvent these behaviors as individuals with ASD grow older. More research is needed regarding the issues addressed in this study.

## Figures and Tables

**Table 1:** Comparisons of participants’ sex and age.

	SIB	Aggression	SIB/Aggression	Neither Behavior
Males	10.3%	17.4%	19.6%	52.6%
	(*n* = 196)	(*n* = 331)	(*n* = 373)	(*n* = 1000)
Females	14.1%	12.5%	21.2%	52.3%
	(*n* = 53)	(*n* = 47)	(*n* = 80)	(*n* = 197)
Under 3 years old	1.9%	23.1%	23.1%	51.9%
	(*n* = 1)	(*n* = 12)	(*n* = 12)	(*n* = 27)
Between 3 and 4 years of age	6.4%	15.8%	14.0%	63.7%
	(*n* = 11)	(*n* = 27)	(*n* = 24)	(*n* = 109)
Between 4 and 5 years of age	9.3%	16.2%	19.8%	54.6%
	(*n* = 16)	(*n* = 28)	(*n* = 34)	(*n* = 94)
Between 5 and 6 years of age	6.8%	19.7%	21.4%	52.1%
	(*n* = 16)	(*n* = 46)	(*n* = 50)	(*n* = 122)
Over 6 years	12.4%	16.1%	20.1%	51.4%
	(*n* = 205)	(*n* = 267)	(*n* = 333)	(*n* = 851)

**Table 2:** Results for only self-injurious behavior (SIB) and SIB and aggression.

	SIB	Aggression	SIB/Aggression	Neither Behavior	Chi-Square Results
Have you child had a seizure? Yes.	22.8%	16.9%	20.8%	14.0%	*χ*^2^ = 23.18
	(*n* = 246)	(*n* = 372)	(*n* = 447)	(*n* = 1176)	*p* = 0.00004
(Age 3–5) Does the child sometimes whirls himself like a top? Yes, does this often.	24.3%	19.5%	24.4%	14.1%	*χ*^2^ = 44.24
	(*n* = 243)	(*n* = 374)	(*n* = 445)	(*n* = 1197)	*p* < 0.00001
(Before age 5) Can the child understand what you say to him, judging from his ability to follow instructions or answer you? Very little or no understanding.	11.0%	9.4%	11.7%	6.6%	*χ*^2^ = 20.92
	(*n* = 245)	(*n* = 380)	(*n* = 446)	(*n* = 1188)	*p* = 0.00011
During the child’s first year, did he seem to be unusually intelligent? Suspected lower than average intelligence.	13.4%	6.9%	15.3%	6.9%	*χ*^2^ = 16.51
	(*n* = 246)	(*n* = 377)	(*n* = 450)	(*n* = 1194)	*p* = 0.00089
Is he/she often anxious or nervous? Yes.	64.9%	53.4%	64.3%	48.4%	*χ*^2^ = 57.68
	(*n* = 245)	(*n* = 371)	(*n* = 446)	(*n* = 1176)	*p* < 0.00001
What is the average amount of time that your child sleeps in a 24-h period? Less than 8 h.	23.4%	12.8%	29.4%	16.4%	*χ*^2^ = 55.17
	(*n* = 247)	(*n* = 376)	(*n* = 448)	(*n* = 1184)	*p* < 0.00001
Seems not to feel pain? True and Very true.	66.1%	56.2%	68.1%	56.5%	*χ*^2^ = 52.36
	(*n* = -245)	(*n* = 377)	(*n* = 448)	(*n* = 1191)	*p* < 0.00001

**Table 3:** Results for SIB and aggression.

	SIB	Aggression	SIB/Aggression	Neither Behavior	Chi-Square Results
(Age 3–5) Is the child destructive? Yes, this is definitely a problem.	23.1%	24.9%	44.9%	6.8%	*χ*^2^ = 319.99
	(*n* = 247)	(*n* = 378)	(*n* = 450)	(*n* = 1197)	*p* < 0.00001
(Age 3–5) Is child upset by certain things that are not “right” (like crack in wall, spot on rug, books leaning in bookcase, broken rung on chair, pipe held and not smoked)? Yes, such things often upset him greatly.	33.7%	27.6%	41.9%	20.5%	*χ*^2^ = 84.48
	(*n* = 249)	(*n* = 381)	(*n* = 451)	(*n* = 1191)	*p* < 0.00001

**Table 4:** Results for only SIB, only aggression, and SIB and aggression.

	SIB	Aggression	SIB/Aggression	Neither Behavior	Chi-Square Results
Did the child rock in his crib as a baby? Yes, quite a lot.	10.9%	9.2%	10.8%	6.4%	*χ*^2^ = 77.91
	(*n* = 248)	(*n* = 379)	(*n* = 453)	(*n* = 1195)	*p* < 0.00001
Any reactions to foods (same symptoms each time when the child eats a specific food)? Yes.	38.0%	37.0%	41.6%	31.7%	*χ*^2^ = 16.68
	(*n* = 245)	(*n* = 376)	(*n* = 449)	(*n* = 1177)	*p* = 0.00082

**Table 5:** Results for gastrointestinal distress.

	SIB	Aggression	SIB/Aggression	Neither Behavior	Chi-Square Results
Constipation	5.7%	7.6%	11.0%	22.9%	*χ*^2^ = 21.44
	(*n* = 133)	(*n* = 178)	(*n* = 255)	(*n* = 534)	*p* = 0.00008
Diarrhea	3.3%	5.4%	8.0%	14.9%	*χ*^2^ = 22.37
	(*n* = 770	(*n* = 125)	(*n* = 186)	(*n* = 347)	*p* = 0.00006

**Table 6:** Summary of the results in relation to SIB, aggression, SIB/aggression, and neither behavior ^1^.

	SIB	Aggression	SIB/Aggression	Neither Behavior
Early signs				
Rocked in crib as a baby	X	X	X	
Age 0–1: Suspected low intelligence	X		X	
Age 0–5: Little or no receptive language	X		X	
Age 3–5: Often whirls like a top	X		X	
Age 3–5: Destructive			X	
Age 3–5: Upset by certain things that are not “right”			X	
Had a seizure as a child	X		X	
No context regarding age				
Constipation				X
Diarrhea				X
Often anxious or nervous	X		X	
Reacts to specific food(s)	X	X	X	
Seems not to feel pain	X		X	
Sleep less than 8 h	X		X	

^1^ “X” refers to a relatively higher occurrence rate as compared to the other categories.

## Data Availability

This data can be found here: doi:10.17632/yx82mv9dts.1.
